# The antitumor potential of Interleukin-27 in prostate cancer

**DOI:** 10.18632/oncotarget.1425

**Published:** 2013-12-28

**Authors:** Emma Di Carlo, Carlo Sorrentino, Alessia Zorzoli, Serena Di Meo, Maria Grazia Tupone, Emanuela Ognio, Gabriella Mincione, Irma Airoldi

**Affiliations:** ^1^ Department of Medicine and Sciences of Aging, Section of Anatomic Pathology and Molecular Medicine, “G. d'Annunzio” University, Chieti, Italy; ^2^ Ce.S.I. Aging Research Center, “G. d'Annunzio” University Foundation, Chieti, Italy; ^3^ Laboratory of Oncology, Istituto Giannina Gaslini, Genova, Italy; ^4^ Animal Facility, IRCCS AOU San Martino-IST Istituto Nazionale per la Ricerca sul Cancro, Genova, Italy; ^5^ Department of Experimental and Clinical Sciences, “G. d'Annunzio” University, Chieti, Italy

**Keywords:** prostate cancer, interleukin-27, cytokines, immunotherapy, anti-tumor activity, tumor microenvironment

## Abstract

Prostate cancer (PCa) is of increasing significance worldwide as a consequence of the population ageing. Fragile elderly patients may particularly benefit from noninvasive and well tolerable immunotherapeutic approaches. Preclinical studies have revealed that the immune-regulatory cytokine IL-27 may exert anti-tumor activities in a variety of tumor types without discernable toxicity. We, thus, investigated whether IL-27 may function as anti-tumor agent in human (h) PCa and analyzed the rationale for its clinical application.

*In vitro,* IL-27 treatment significantly inhibited proliferation and reduced the angiogenic potential of hPCa cells by down-regulating the pro-angiogenesis-related genes fms-related tyrosine kinase (FLT)1, prostaglandin G/H synthase 1/cyclooxygenase-1 (PTGS1/COX-1) and fibroblast growth factor receptor (FGFR)3. In addition, IL-27 up-regulated the anti-angiogenesis-related genes such as CXCL10 and TIMP metallopeptidase inhibitor 3 (TIMP3). *In vivo,* IL-27 reduced proliferation and vascularization in association with ischemic necrosis of tumors developed after PC3 or DU145 cell injection in athymic nude mice. In patients' prostate tissues, IL-27R was expressed by normal epithelia and low grade PCa and lost by high tumor grade and stages. Nevertheless, IL-27R was expressed by CD11c^+^, CD4^+^ and CD8^+^ leukocytes infiltrating the tumor and draining lymph nodes.

These data lead to the conclusion that i) IL-27's anti-PCa potential may be fully exploited in patients with well-differentiated, localized IL-27R positive PCa, since in this case it may act on both cancerous epithelia and the tumor microenvironment; ii) PCa patients bearing high grade and stage tumor that lack IL-27R may benefit, however, from IL-27's immune-stimulatory properties.

## INTRODUCTION

Since the incidence of prostate cancer (PCa) increases with age, the number of new cases diagnosed in the Western countries will rise in the foreseeable future as the inevitable consequence of the universal aging of their populations. Less aggressive and more tolerable therapeutic approaches than radical management may thus be preferable particularly for elderly patients.

The recently identified heterodimeric member of the IL-6/IL-12 family of cytokines [1,2] namely interleukin (IL)-27, has revealed potent anti-tumor effects in various tumor models and, importantly, freedom from toxicity in preclinical trials [3]. IL-27 displays anti-tumor activity via different mechanisms [3]. It has been reported to exert anti-proliferative and anti-angiogenic effects by directly acting on cancer cells in melanoma [4,5], B acute lymphoblastic leukemia [6], acute myeloid leukemia [7], B cell lymphoma [8] and multiple myeloma [9]. It also exerts indirect anti-tumor effects driven by its immune-stimulatory activity in melanoma [10], colon carcinoma [11,12], neuroblastoma [13], lung cancer [14], and head and neck squamous cell carcinoma [15]. Studies on IL-27's effects in PCa have so far been carried out both *in vitro* with murine prostate cancer cell lines [16] and *in vivo* with immune-competent murine PCa models [17]. These findings open the perspective to candidate IL-27 as therapeutic agent in PCa patients. We therefore investigated this issue using *in vitro* and *in vivo* models, and analyzing the expression of IL-27 receptor (R) in prostate tissues and draining lymph nodes from PCa patients with different tumor grades and stages.

## RESULTS

### IL-27 inhibits human PCa cell proliferation *in vitro*

Since IL-27 revealed anti-tumor effects in a variety of tumor models, we assessed whether IL-27 may function as anti-tumor agent against human (h) PCa. To this end, in *vitro* and *in vivo* studies were performed using hPCa cell lines.

**Figure 1:**
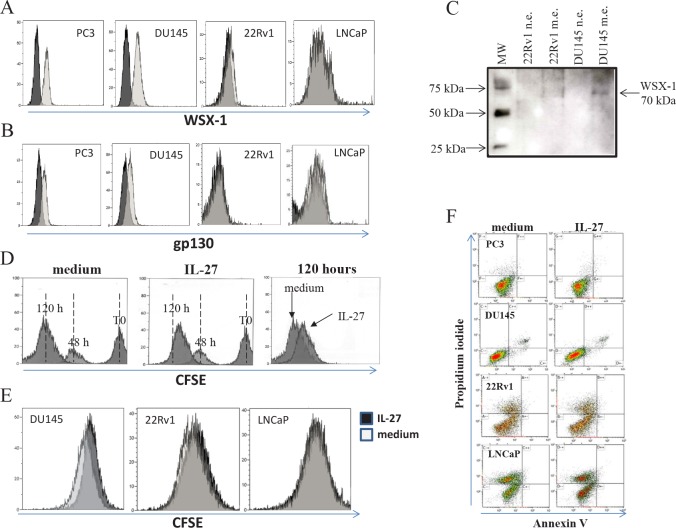
Expression of IL-27R on hPCa cell lines and assessment of IL-27 effects on hPCa cells *in vitro* and *in vivo* Panel A and B. Expression of WSX-1 (Panel A) and gp130 (panel B) was analysed in PC3, DU145, 22Rv1 and LNCaP cells by flow cytometry. Open profile: WSX-1 (panel A) and gp130 (panel B) staining. Dark profile: isotype matched mAb staining. Experiments were performed at least in triplicate. Panel C. Western blot analyses of WSX-1 in nuclear extracts (n.e.) and membrane extracts (m.e.) from 22Rv1 and DU145 cells. Nuclear extracts were used as negative controls. A specific 70 kDalton (Da) band, corresponding to WSX-1 protein, was observed in membrane but not nuclear extracts obtained from DU145 cells. Panel D. IL-27 inhibits PC3 cell proliferation *in vitro*, as assessed by CFSE staining. PC3 cells were cultured for 48 and 120 hours with medium alone (left panel) or in the presence of 100 ng/ml hrIL-27 (middle panel). Flow cytometry analyses showed that IL-27 inhibited PC3 cell proliferation after 120 hours of treatment (middle and right panel), as indicated by the higher CFSE intensity in hrIL-27 treated PC3 cells compared to untreated cells at this time point (right panel). Panel E. Flow cytometry analyses showed that IL-27 inhibited DU145 (left panel) cell proliferation after 120 hours of treatment, as indicated by the higher CFSE intensity in hrIL-27 treated cells (dark profile) compared to untreated cells at this time point (light profile). IL-27 did not affect 22Rv1 (middle panel) or LNCaP (right panel) cell proliferation at the same time point. Panel F. IL-27 did not induce apoptosis after 120 hours of treatment in PC3, DU145, 22Rv1 or LNCaP cells.

We first assessed the expression of both chains of IL-27R, i.e. gp130 and WSX-1 [18,19], in human PC3, DU145, LNCaP and 22Rv1 cells, by flow cytometry. Human PC3 and DU145 cells, but not LNCaP and 22Rv1 cells, express both WSX-1 and gp130 chains at surface level (Figure 1A and B, respectively), thus indicating that PC3 and DU145 cells may respond to IL-27. The expression of WSX-1 in DU145 cells has been confirmed by western blot (Figure 1C).

To assess the ability of IL-27 to affect hPCa cell proliferation and apoptosis, cells were cultured with or without human recombinant (hr) IL-27 for 120 hours and an aliquot of these cells was harvested every 24 hours for flow cytometry with CFSE intracellular staining, cell count or annexin V/PI staining.

hrIL-27 inhibited PC3 cell proliferation after 120 hours (Figure 1D) as shown by the higher CFSE intensity in hrIL-27 treated cells at this time point (Figure 1D). A similar behavior was observed in DU145 (Figure 1E left panel), but not in 22Rv1 or LNCaP (Figure 1E, middle and right panel, respectively), treated with hrIL-27.

Analysis of apoptotic cells, identified by flow cytometry as Annexin V^+^/PI^+^ cells, revealed that hrIL-27 did not induce any significant apoptotic effect in any cell line, irrespective of the time of treatment. One representative experiment is shown in Figure 1F. Finally, direct cell counting of trypan blue stained PC3 and DU145 cells using an automated cell countess, revealed that 120 hour of treatment with hrIL-27 caused a reduction in the absolute number, but not in the viability measured as ratio between alive and dead cells, of both PC3 and DU145 cells. In detail, the absolute number of alive PC3 cells treated with hrIL-27 was 1.15±0.2×10^6^, that of untreated cells was 2.08±0.3×10^6^, thus indicating that hrIL-27 treated PC3 cells were 55.1% of untreated cells. Similarly, the absolute number of alive DU145 cells treated with hrIL-27 was 1.3±0.2 × 10^6^, that of untreated cells was 1.8±0.3×10^6^, thus indicating that hrIL-27 DU145 treated cells were 73% of untreated cells. Taken together, IL-27 exerts, *in vitro,* an anti-proliferative but not pro-apoptotic effect against human PCa cell lines that express the complete corresponding receptor.

### hPCa xenograft responds to hrIL-27 through a decreased tumor cell proliferation and tumor vascularization

We next tested whether hrIL-27 is effective *in vivo* on hPCa tumor growth. To this end, PC3 or DU145 cells were injected subcutaneously (s.c.) in athymic nude mice that were subsequently treated with hrIL-27.

**Figure 2:**
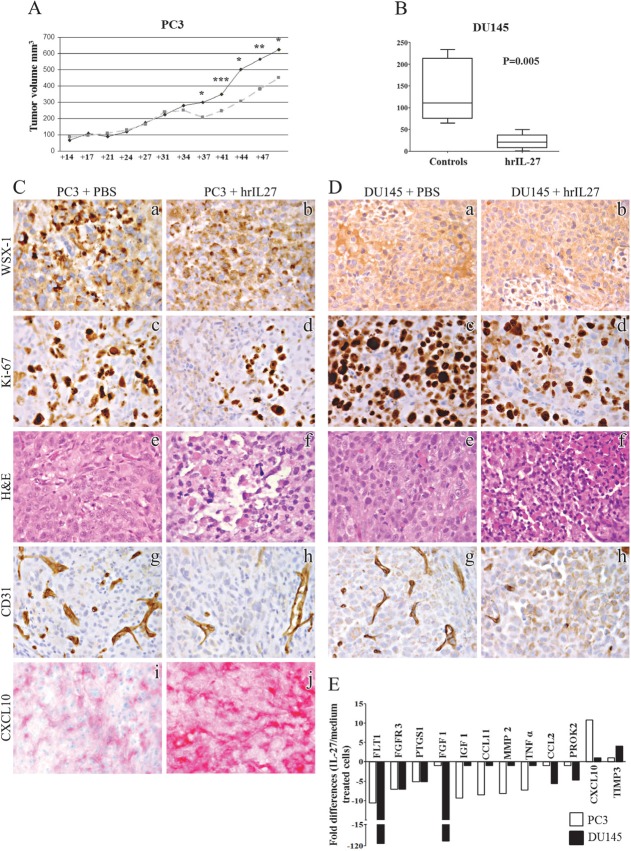
Inhibition of human PC3 and DU145 cell growth *in vivo* by IL-27 treatment Panel A. Tumor growth curve from day 14 to day 51 after PC3 s.c. cell injection into athymic nude mice was obtained by measuring tumor volume *in situ* using a caliper. Dark line represents growth curve (volume) of tumors formed in mice injected with PC3 cells and treated with PBS (controls). Grey light line represents growth curve of tumors formed in mice injected with PC3 cells and treated with hrIL-27. Asterisks mean significant differences (see results section). Panel B. Volume of tumor masses developed in mice injected with the DU145 cell line and subsequently treated with PBS (controls) or hrIL-27. Panel C and D. Morphological and immunohistochemical features of PC3 (C) and DU145 (D) tumor masses grown in hrIL-27 or PBS treated mice. Expression of WSX-1 was preserved *in vivo* by tumor developed after s.c. injection of PC3 or DU145 cells in both control (a) and hrIL-27 treated (b) mice. The proliferative activity of PC3 tumors grown in PBS treated animals (c) was considerably reduced in tumors from hrIL-27 treated animals (d). The histologic feature of poorly differentiated tumor with a solid growth pattern (e) and endowed with a well-developed vascularization (g) observed in PBS treated animals, was heavily compromised in tumors from hrIL-27 treated animals which showed multiple foci of ischemic necrosis (f) along with a deficient vascular supply (h). PC3 tumors revealed a strong expression of the anti-angiogenic chemokine IP-10/CXCL10 (j), which, on the contrary, was barely detected in tumors from PBS treated animals (i). (a-j: x400). Panel E. Expression profile of genes involved in angiogenic pathways in PC3 (white bars) and DU145 (black bars) cells treated with hrIL-27. Pooled results ± SD from two experiments performed in duplicate are shown. Histogram represents fold differences of individual mRNA between cells cultured in presence or absence of hrIL-27.

The volume of tumors developed after PC3 cell inoculation did not differ significantly between hrIL-27 treated and control mice until day 34 (Fig. 2A). Significant differences were apparent at days 37 (*P* = 0.0192, mean tumor volume, mtv, in treated mice *vs* controls: 212 mm^3^ *vs* 306 mm^3^), 41 (*P* = 0.0005, mtv in treated mice *vs* controls: 245 mm^3^ *vs* 347 mm^3^), 44 (*P* = 0.0379, mtv in treated mice *vs* controls: 305 mm^3^ *vs* 508 mm^3^), 47 (*P* = 0.0037, mtv in treated mice *vs* controls: 380 mm^3^ *vs* 564 mm^3^) and 51 (*P* = 0.0473, mtv in treated mice *vs* controls: 451 mm^3^ *vs* 625 mm^3^).

Similarly, tumors developed from DU145 cell injection were significantly smaller than those developed in control mice starting from day 9 (*P* = 0.0203, mtv in treated mice *vs* controls: 19.33 mm^3^ *vs* 106.4 mm^3^, Fig. 2B). At day 14 tumors were measured, removed from animals and used for histological and immunohistochemical studies.

Immunohistochemical analyses of PC3 and DU145 tumor masses revealed that both PC3 (Fig. 2C, a and b) and DU145 (Fig. 2D, a and b) cells express WSX-1 *in vivo* with no appreciable difference between control and hrIL-27-treated tumors. hrIL-27 significantly decreased their proliferation (*P* < 0.05), as shown by Ki-67 immunostaining (Table 1 and Fig. 2C, c and d; Fig. 2D, c and d), and induced multiple foci of ischemic necrosis (Fig. 2C, e and f; Fig. 2D, e and f), as assessed by histology, in association with a defective microvascular supply, as shown by CD31 immunostaining (Fig. 2C, g and h; Fig. 2D, g and h and Table1).

**Table 1:** Immunohistochemical analyses of tumors developed after subcutaneous injection of PC3 or DU145 cells in athymic nu/nu mice treated with PBS or hrIL-27

	PC3						DU145					
	PBS			hrIL-27			PBS			hrIL-27		
Proliferation index ^a^	64.5	±	9.2%	40.3	±	8.5%*	84.0	±	11.0%	63.0	±	8.5%*
Vessel count ^a^	13.5	±	4.0	4.3	±	2.5*	12.4	±	3.6	3.7	±	2.2*

a Microvessel and cell count performed at x400 in a 0.180 μm2 field. At least 3 samples (1 sample/tumor growth area) and 8-10 randomly chosen fields/sample were evaluated. Results are expressed as mean ± SD of CD31 positive microvessels per field or Ki-67 positive cells/number of total cells evaluated on formalin-fixed sections by immunohistochemistry.

* Values significantly different (P < 0.05) from corresponding values in tumors developed in PBS-treated mice.

### IL-27 modulates angiogenesis related gene expression in PC3 cells *in vitro*

The histopatological demonstration of IL-27's anti-angiogenic effects *in vivo*, led us find out whether it affects the expression of genes involved in the angiogenic pathways in PC3 and DU145 cells *in vitro*, by PCR Array. As shown in Figure 2E, hrIL-27 significantly down-regulated, in both cell lines, pro-angiogenic genes such as vascular endothelial growth factor receptor (VEGFR)1/FLT1 (10.6 fold down-regulation in PC3 and 108.8 in DU145 cells), fibroblast growth factor receptor 3 (FGFR3, 7 fold down-regulation in both PC3 and DU145 cells) and prostaglandin G/H synthase 1 (PTGS1, 5.1 fold down-regulation in both cell lines). Further pro-angiogenic molecules were found to be modulated by hrIL-27 exclusively in PC3 or in DU145 cells. In the former, hrIL-27 down-regulated mRNA expression of insulin-like growth factor (IGF)1 (9 fold down-regulation), C-C motif chemokine (CCL)11/eotaxin-1 (8.5 fold down-regulation), matrix metalloproteinase (MMP)2 (8 fold down-regulation), tumor necrosis factor (TNF) alpha (7 fold down-regulation) and cyclooxygenase (COX) −1 (5 fold down-regulation). By contrast, in DU145 cells, hrIL-27 down-regulated mRNA expression of fibroblast growth factor (FGF)1 (95.4 fold down-regulation), chemokine (C-C motif) ligand 2 (CCL2) (5.6 fold down-regulation) and prokineticin (PROK)2 (4.7 fold down-regulation). Moreover, hrIL-27 up-regulated mRNA expression of the anti-angiogenic molecule interferon gamma-induced protein (IP-10/CXCL)10 (11 fold up-regulation) in PC3 cells and TIMP metallopeptidase inhibitor 3 (TIMP3) (4.1 fold up-regulation) in DU145 cells (Fig. 2E). Immunohistochemistry performed on PC3 tumors grown in PBS or hrIL-27-treated mice disclosed a stronger IP10/CXCL10 expression in those from the latter group as evidence of its involvement in the anti-angiogenic effect displayed by IL-27 *in vivo* (Fig. 2C, i and j).

### WSX-1 is lost in high grade and advanced stages PCa, but expressed by tumor infiltrating leukocytes (TIL) and endothelial cells

**Figure 3:**
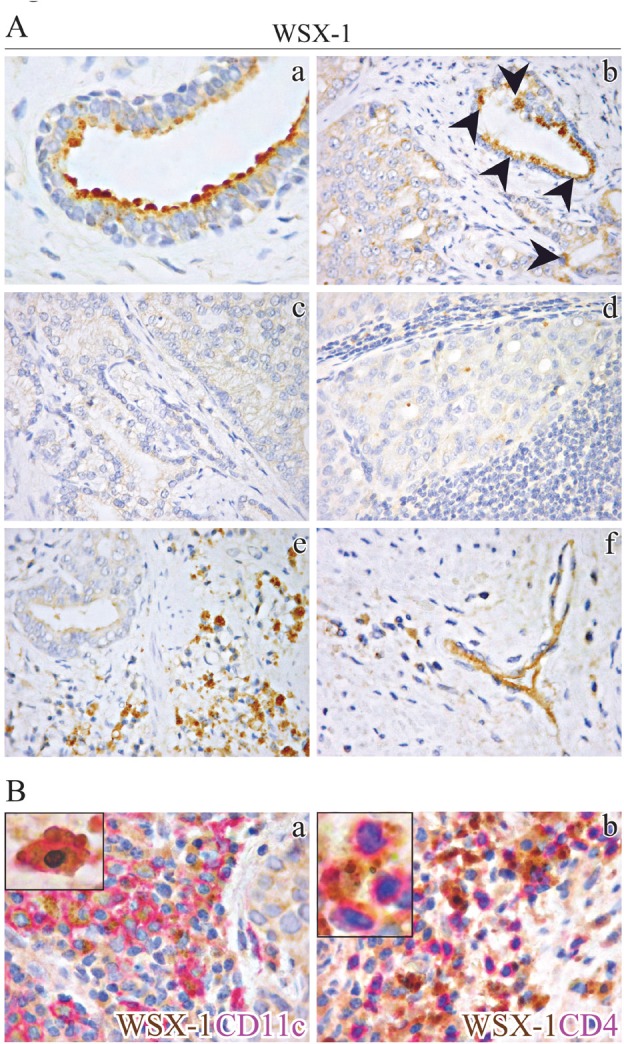
Immunohistochemical localization of WSX-1 in normal and neoplastic human prostate tissues (A) WSX-1 expression in normal prostate, primary PCa and lymph node metastasis. WSX-1 was expressed by luminal secretory epithelia in normal prostate (a) and low-grade PCa foci (indicated by arrowheads in b), and usually lost in primary PCa (c) and lymph node metastasis (d), but could be found on immune cells infiltrating PCa stroma (e) and in microvessel endothelia (f). (a: x630; b-d: x400). (B) WSX-1 expression in PCa stroma. Double immunohistochemistry located WSX-1 expression (brown) on cells endowed with CD11c^+^ (fuchsia) (a) and CD4^+^ (fuchsia) (b) phenotype, as better shown in the insets (upper left sides). (a, b: x400; insets: x1000).

To determine whether hPCa patients could benefit from IL-27's anti-tumor effects, we next immunohistochemically evaluated expression and distribution of IL-27R in prostate tissue sections from PCa patients following radical prostatectomy (RP). Since expression of glycoprotein gp130 has been reported in hPCa epithelia [18], we assessed WSX-1 expression in normal prostate tissue (from both PCa and control patients) and PCa. It was detected in normal luminal secretory epithelial cells (Fig. 3A, a) and may be found in cells with leukocyte features homing normal prostate stroma. In the cancerous samples, WSX-1 was still detectable in most well differentiated PCa foci (Gleason ≤ 3) (Fig. 3A, arrowheads in b), but was usually lost in the vast majority of poorly differentiated PCa foci (Gleason > 3) and in lymph node metastatic nests (Fig. 3A, c and d). By contrast, WSX-1 was frequent within PCa stroma, in infiltrating cells with leukocyte features, in small vessel endothelia (Fig. 3A, e and f), and in immune cells homing prostate draining lymph node microenvironment. This pattern of WSX-1 expression in the tumor microenvironment was observed in both low and high grade PCa. Double immunostaining exactly located WSX-1 expression, in the primary tumor and draining lymph nodes, on immune cells endowed with CD11c^+^ and CD4^+^ phenotype, to a lesser extent (Fig. 3B, a and b), and rarely on CD8^+^ phenotype.

## DISCUSSION

PCa is an age-related malignancy that tends to develop in men over the age of fifty. Longer life expectancy will make it a substantial public health concern in the near future. Its slow growth, however, means that many men never display cancer-related symptoms, and eventually die of other causes. Thus, overtreatment and related side effects may be a growing problem, especially among older patients.

An alternative option may be represented, in selected cases, by a rational and well tolerable immunotherapeutic approach. The lack of toxicity revealed in preclinical trials [3] along with the powerful anti-cancer effects demonstrated in different tumor types [4-17] have driven our attempt to explore IL-27 as a candidate anti-PCa agent.

IL-27 is a heterodimeric cytokine composed of a helical protein, IL-27p28, and a soluble cytokine receptor-like component, EBI3 [19]. Through engagement of its cognate receptor, IL-27 can activate an intra-cellular signaling cascade. WSX-1 together with gp130 constitutes a functional signal-transducing receptor, IL-27R, for IL-27 [20].

Assessment of gp130 and WSX-1 expression in human PC3, DU145, 22Rv1 and LNCaP cancer cells revealed that PC3 and DU145 cell lines express complete IL-27R on the surface. This may explain why these cells only show a significant biological response to IL-27 *in vitro* in the form of a significant reduction in cell proliferation and a prominent change in the cancer cell angiogenic program. IL-27 caused, in both cell lines, a significant down-regulation of a series of anti-angiogenesis related genes namely FGFR3 [21], PTGS1 [22] and, particularly, FLT1 [23], which has been reported to be firmly and strongly expressed in hPCa [24,25] and involved in cancer cell proliferation via autocrine VEGF signaling [26]. Some anti-angiogenesis related genes were essentially down-regulated in PC3 cells, i.e. MMP2 [27], TNF-alpha [28], CCL11 [29] and the potent pro-angiogenic factor IGF1 (9 fold down-regulation) which is deeply involved in prostate carcinogenesis [30,31] and identified as autocrine proliferation stimulus for hPCa cells [32]. Whereas others, such as PROK2 [33] and CCL2 [34] were down-regulated just in DU-145 cells.

The only pro-angiogenic genes found up-regulated, and substantially, were that coding the chemokine IP-10/CXCL10 [35] which was also strongly expressed *in vivo* by PC3 tumor from IL-27 treated animals, and the metalloproteinase inhibitor, TIMP3 [36], in DU145 cells.

*In vivo* studies revealed that IL-27 hampered PC3 tumor growth in athymic nude mice and significantly reduced tumor volume after more than one month of treatment. Such IL-27 anti-tumor activity was related to the direct inhibition of PC3 angiogenic program towards an anti-angiogenic phenotype that allows consistent subversion of the host derived microvascular network resulting in a heavily compromised architecture and rarefied endothelial branching. Consistently*, in vivo* studies using the DU145 cells revealed a significant and more rapid inhibition of tumor growth by IL-27 treatment than that observed using the PC3 cells. This is conceivably due to the higher surface expression of IL-27R in DU145 cells compared to PC3 cell line.

The imperative need to use immunodeficient mice for xenograft experiments makes it impossible to assess involvement of immune cells, particularly the lymphoid lineage, in the *in vivo* effects of IL-27. Zolochevska et al. reported that in immunocompetent mice, IL-27 gene therapy leads to the recruitment, in prostate tumors, of CD3^+^CD8^+^ cells, to a decrease in Gr1^+^CD11b^+^CD124^+^ and Gr1^+^CD11b^+^ cells, putative myeloid derived suppressor cells, and CD4^+^CD25^+^Foxp3^+^ cells, potential Treg population [17]. These results suggest that, in PCa, IL-27 may function as immuno-stimulatory mediator which boosts anti-tumor effector cells and restrains immune suppressor mechanisms.

Analyses of prostate tissues obtained from both low and high grade PCa patients show that WSX-1 is expressed mainly by CD11c^+^ myeloid dendritic cells and monocytes/macrophages population, CD4^+^ and, to a lesser extent, by CD8^+^ immune cells infiltrating PCa stroma or homing prostate draining lymph nodes and also by endothelial cells, thus suggesting that exogenously administered IL-27 could affect the local tumor microenvironment. Co-expression of gp130 and WSX-1 (IL-27R) has been documented, in fact, in a variety of immune cell types including activated dendritic cells and monocytes, with greatest expression in the lymphoid lineage, particularly in T cells, and also in endothelial and epithelial cells [20, 37-39].

Taken as whole, we demonstrate for the first time that i) IL-27 exerts direct anti-proliferative and anti-angiogenic effects on PCa cells expressing IL-27R both *in vitro* and in pre-clinical models; ii) in humans, IL-27R is expressed in well-differentiated and localized PCa, but not in high grade and advanced stages PCa; iii) in the tumor stroma, immune and endothelial cells express IL-27R.

Therefore, it is expected that PCa patients bearing high grade and stage tumor may benefit from the IL-27 immunostimulatory [3] and anti-angiogenic [5] effects on the tumor microenvironment, whereas, the full anti-cancer potential of IL-27 may be exploited in patients who are diagnosed with well-differentiated, slow growing and localized PCa. This vast category of patients may never need treatment and only an “active surveillance” is currently recommended by urologists, while IL-27 based immunotherapy could be an effective weapon against unexpected tumor progression.

## MATERIALS AND METHODS

### Ethics Statement

Investigation has been conducted in accordance with the ethical standards and according to the Declaration of Helsinki and according to national and international guidelines and has been approved by the authors' institutional review board.

### Patients and samples

Normal prostates were obtained from 12 untreated patients aged 57-63 following prostatectomy for bladder cancer (control patients). They were histologically negative for PCa or benign prostatic hyperplasia. PCa were from 40 patients aged 64-74 following RP for histologically verified adenocarcinomas at biopsy. Both normal and cancer specimens, and pelvic lymph nodes from each PCa patient, were collected.

Preoperative androgen deprivation had not been employed. PCa samples were graded as Gleason score (Gs) 5, *n*=6; Gs 6, *n*=7; Gs 7, *n*=14; Gs 9, *n*=8; Gs 9, *n*=5 and staged as pT2 (organ confined cancer), *n=*24 (6 T2a, 10 T2b, 8 T2c); pT3 (capsular penetration), *n=*16 (6 T3aN0M0, 5 T3aN1M0, 2 T3bN0M0, 3 T3bN1M0).

Tissue samples were fixed in 4% formalin and embedded in paraffin for histological and immuno-histochemical analyses. Written informed consent was obtained from patients. The study has been approved by the Ethical Committee for Biomedical Research of the Chieti University and Local Health Authority No. 2 Lanciano-Vasto-Chieti in PROT 1945/09 COET of 14/07/2009, and performed in accordance with the principles outlined in the Declaration of Helsinki.

### Cell culture, antibodies, reagents, flow cytometry and western blot

The human PC3, DU145, 22Rv1 and LNCaP PCa cell lines (LGC Standards, Teddington, UK) were cultured in RPMI 1640 with 10% FCS (Seromed-BiochromKG, Berlin, D). hrIL-27 (R&D System, Minneapolis, MN, USA) was used at 100 ng/ml following titration experiments. The expression of both chains of IL-27R was analyzed using FITC conjugated anti-gp130 (R&D Systems) and PE-conjugated anti-WSX-1 (R&D systems) mAbs. Isotype-matched antibodies of irrelevant specificity (Caltag, Burlingame, CA, USA) were used as controls. Cells were run on a Gallios flow cytometer (Beckman Coulter, Brea, CA, USA) and at least 10^4^ events were acquired. Data were analyzed with Kaluza analysis software (Beckman Coulter).

Western blot was performed using membrane and nuclear extracts (Qproteome cell compartment kit from Qiagen, Hilden, Germany) obtained from 5×10^6^ DU145 or 22Rv1 cells. We choose to use these cell lines since WSX-1 was expressed at high level in DU145 cells whereas it was virtually absent in 22Rv1 cells. Standard procedures were followed for SDS page and blotting. Twenty μg of proteins were loaded into 10% SDS poli-acrylamide gel and 1μg/ml rabbit anti human WSX-1 polyclonal antibody (Novus Biologicals) was used. After washings, an anti-rabbit horseradish peroxidase (HRP) link antibody (1:5000) from Cell Signaling was used as secondary antibody. The ECL select western blotting detection reagent from Amersham was added to visualize proteins.

### Cell proliferation and apoptosis assay

The human PC3 and DU145 prostatic carcinoma cell lines were cultured for 24, 48, 72, 96 and 120 hours with or without 100 ng/ml hrIL-27. Cells were incubated with 2 μM Carboxy-Fluorescein diacetate Succinimudyl Ester (CFSE) in RPMI 1% FCS for 15 minutes at 37 °C, washed in RPMI 10%FCS, plated and analyzed by flow cytometry at the above-mentioned time points. In addition, cells were counted every 24 hours after staining with trypan blue using the authomated cell countess from Life Technologies (Carlsbad, CA, USA).

Apoptosis was assessed using the Annexin V-FITC Kit from Immunostep (Salamanca, E), and apoptotic cells were identified as Annexin V^+^/PI^+^ cells by flow cytometry.

### Mouse studies

Four- to six-week-old athymic nude mice (Harlan Laboratories, Udine, Italy) were housed under specific pathogen-free conditions. All procedures were performed in accordance with the current National and International regulations (EU Directive 2010/63/EU).

Two groups of 8 animals were injected s.c. with 6×10^6^ PC3 cells. One group was treated s.c. with 2 weekly doses of hrIL-27 (1 mg/mouse/dose) starting from 2 days after tumor cells injection. The other group was injected with PBS (controls) according to the same time schedule. Tumors were measured *in situ* every three days with a caliper starting from day fourteen, when palpable masses were developed. Mice were sacrificed at day fifty-four when signs of poor health were evident. Two groups of six PBS or hrIL-27 treated animals were sacrificed 37 days after PC3 cell injection.

*In vivo* studies with the DU145 cells were performed by injecting 6×10^6^ cells s.c. in two groups of eight athymic nude mice. As of PC3 cell line, one group was used as controls and the other one was treated with hrIL-27 using the same time schedule reported above. Tumors were measured *in situ* every three days using a caliper starting from day three, when palpable masses were developed. Mice were sacrificed at day 14 when signs of poor health were evident.

Tumors from mice injected with PC3 and with DU145 cells were removed, formalin-fixed and paraffin-embedded or snap-frozen in liquid nitrogen for histological and immuno-histochemical analyses.

### PCR Array

Total RNA was extracted with the RNeasy micro kit (Qiagen) from PC3 or DU145 cells cultured overnight with 100 ng/ml hrIL-27 or medium alone. Contaminant genomic DNA was removed by Dnase treatment (Qiagen). RNA was retrotranscribed with the RT^2^First Strand cDNA Synthesis kit (SABioscience, Frederick, MD, USA). Human Angiogenesis (code #PAHS-024Z) RT^2^PCR Array and RT^2^Real-Time SYBR Green/ROX PCR Mix were from SABioscience. PCR was done on an ABI Prism 7700 Sequence Detector (Applied Biosystems, Foster City, CA, USA). Gene expression of hrIL-27-treated and control samples was analyzed separately in different PCR Array plates. Results for each plate were normalized on the median value of a set of housekeeping genes. Changes in gene expression between hrIL-27 treated and control samples were then calculated using the ΔΔCt formula. Results (obtained in duplicate) were pooled and analyzed with the software provided by the manufacturer. A significant threshold of 4-fold change in gene expression corresponded to *P* < 0.001.

### Morphologic and immunohistochemical analyses

For histology, paraffin-embedded samples were sectioned at 4 μm and stained with hematoxylin and eosin.

For immunohistochemistry on the formalin-fixed, paraffin-embedded samples, sections were deparaffinized and subsequently incubated for 30 minutes with primary antibodies listed in Table 2. For immunohistochemistry on frozen samples, cryostat sections were fixed in acetone for 10 minutes and, after washing in PBS/Tween-20, incubated with mouse anti-human IP10/CXCL10 antibody (Abcam). Immune complexes were detected using the Bond Polymer Refine Detection Kit according to the manufacturer's protocol (Leica Biosystems, Wetzlar, Germany), then sections were counterstained with hematoxylin and eosin.

**Table 2:** Antibodies used in immunostaining

Antibody	Clone	Origin	Dilution	Source
On human tissue				
CD4	4B12	Mouse	1:50	Leica Biosystems (Newcastle Upon Tyne, UK)
CD8	C8/144B	Mouse	1:80	Dako (Glostrup, DK)
CD11c	EP1347Y	Rabbit	1:250	Abcam (Cambridge, UK)
IP10/CXCL10*	6D4	Mouse	1:20	Abcam
Ki-67	MIB-1	Mouse	1:100	Dako
WSX-1		Rabbit	1:100	Novus Biologicals (Cambridge, UK)
On murine tissue				
CD31	SZ31	Rat	1:20	Dianova (Hamburg, Germany)

* Used on frozen sections.

For WSX-1/CD4, WSX-1/CD8 and WSX-1/CD11c double stainings on formalin-fixed paraffin-embedded samples, sections were deparaffinized, treated with H_2_O_2_/3% for 5 minutes to inhibit endogenous peroxidase, and then washed in H_2_O. The slices were then incubated for 30 minutes with the first primary antibody (anti-WSX-1) followed by detection with the Bond Polymer Refine Detection Kit (Leica Biosystems) according to the manufacturer's protocol. Then, sections were incubated for 30 minutes with the second primary antibody (anti-CD4, anti-CD8 and anti-CD11c) followed by detection with the Bond Polymer Refine Red Detection Kit (Leica Biosystems) according to the manufacturer's protocol.

### Statistical analysis

Tumor volumes were reported in mm^3^ *versus* time. Data of microvessel density and Ki-67 positive cell counts were reported as mean ± standard deviation (SD). Differences in tumor volume, vessel counts or proliferating cell percentage between tumors from hrIL-27 or PBS treated mice were assessed by Student's t-test. The Mann–Whitney U probability test was used to examine the association between IP-10/CXCL10 staining and the hrIL-27 treatment. The SPSS software, version 11.0 (IBM, Armonk, NY, USA) was employed, with *P* < 0.05 as the significance cut-off.
